# Executive functioning is preserved in healthy young adults under acute sleep restriction

**DOI:** 10.5935/1984-0063.20180029

**Published:** 2018

**Authors:** Thais Schaedler, Jefferson Souza Santos, Roberta Almeida Vincenzi, Sofia Isabel Ribeiro Pereira, Fernando Mazzilli Louzada

**Affiliations:** 1 Federal University of Paraná, Department of Physiology - Curitiba - Paraná - Brasil.

**Keywords:** Executive Functions, Impulsive Behavior, Sleep Deprivation, REM sleep

## Abstract

**Objectives:**

This study aimed to evaluate if a partial morning or evening sleep restriction protocol could affect executive functioning in healthy young adults.

**Methods:**

Participants were assigned to one of three groups: control (n=18), in which participants maintained their habitual sleep/wake cycle; morning restriction (n=17), in which volunteers terminated sleep approximately three hours earlier than the usual on the experimental night, and evening restriction (n=13), in which volunteers initiated sleep approximately three hours later than the usual on the experimental night. On the day of the experiment, they performed the Stroop Test, the Go-NoGo Test and the Iowa Gambling Task (IGT).

**Results:**

When compared to the control group, neither morning nor evening sleep-restricted individuals displayed any significant deficits in: a) selective attention as assessed by the interference index (H=3.38; *p*=0.18) and time to performed the interference card (H=2.61; *p*=0.27) on the Stroop test; b) motor response inhibition as assessed by number of false alarms (H=0.8; *p*=0.67) on the Go-NoGo Test; and c) in decision-making as assessed by total won (H=2.64; *p*=0.26) and number of selected advantageous cards (H=4.43; *p*=0.11) on the IGT.

**Conclusion:**

These findings suggest that the ability to pay attention, inhibit a motor response and make decisions is preserved following approximately 3 hours of sleep restriction, regardless of its timing (in the morning or in the evening).

## INTRODUCTION

The advent of electricity in the late XIX century enabled the emergence of new production models and the gradual development of electronic devices such as televisions, computers and more recently, smartphones. This technological leap affected the sleep/wake cycle by increasing light exposure^1^ and waking hours in order to work, study or engage in one of the many new entertainment options^2^^,^^3^. Consequently, the prevalence of chronic sleep restriction and sleep disorders has increased in recent years^4^^,^^5^.

Sleep architecture is cyclic, alternating between two main stages: REM (Rapid Eye Movement) and NREM (Non-Rapid Eye Movement) sleep. NREM sleep is divided into NREM 1, NREM 2 and NREM 3 or slow wave sleep (SWS). In healthy young adults, a typical night of sleep is characterized by a high concentration of SWS in the first half of the night and a prevalence of REM sleep in the second one^6^. The modifications in sleep architecture caused by sleep restriction depend on when it was enforced (the first or second half of the night) and include an increase in REM and SWS duration the following night^7^^-^^9^.

In agreement with the assumptions from the dual hypothesis, studies from the beginning of this century using the half-night paradigm or selective sleep stage deprivation have shown that SWS predominantly benefits declarative memory, while REM sleep is more important for emotional and complex (encompassing both motor and declarative components) memories^10^. Intact memory faculties (including its working, motor and emotional subdivisions) are key to the proper running of executive functions (EF)^11^.

The concept of EF is generally defined as an umbrella term, which comprises cognitive processes that allow subjects to direct behaviors, evaluate them and adjust to environmental changes contributing to problem solving. Impairments of those abilities could be related to disorders that present symptoms suggestive of increased impulsiveness^11^.

Impulsive behavior could be characterized by an inability to wait, a tendency to act without forethought, insensitivity to consequences and an inability to inhibit inappropriate behaviors^12^^-^^14^. Due to this concept’s complexity, impulsiveness has been divided into different dimensions. In the present work, we adopted the three dimensions of impulsive behavior proposed by Patton et al.^15^: attentional, motor and cognitive. Changes in the attentional dimension are characterized by impairment of focus and attention; changes in the motor dimension are characterized by an inability to suppress unwanted motor responses^16^ and modifications in the cognitive dimension are characterized by the involvement in actions with high risk of punishment or loss in order to achieve a reward^17^.

It is well known that the level of alertness can influence EF^18^. Regarding selective attention, as assessed by the Stroop test, the literature shows that a 36h-wakefulness exposure protocol did not affect performance^19^, but a 40h-wakefulness protocol increased the number of errors and time to respond to the interference card, suggesting an attentional impairment^20^. Performance on tasks evaluating motor response inhibition, such as the Go-NoGo Test, is impaired after total sleep deprivation (TSD), as evidenced by a decrease in motor response retention capacity^21^ and by a reduction in the number of correct answers^22^.

In addition, there is a relationship between slow reaction time on the Go-NoGo test and worst subjective sleep quality^23^. A 75h-period of sleep deprivation impairs decision-making abilities on the Iowa Gambling Task (IGT)^24^. TSD has also been associated with increased sleepiness and impaired decision making^25^sleep loss was positively associated with RTB, and there was evidence that changes in sleep loss are causally related to changes in RTB. One possible mediator of the relationship between sleep loss and RTB was reduced functioning of the ventromedial prefrontal cortex (VMPFC). Studies using positron emission tomography have shown that TSD in healthy adults leads to a decrease in metabolic activity throughout the prefrontal cortex (PFC)^26^, the major brain region recruited while performing these behavioral tests^27^^-^^29^.

In summary, there is a strong body of evidence to support the claim that TSD affects EF. Although acute sleep restriction occurs much more often than TSD, relatively few studies have examined its effect on executive functioning. Therefore, the present study aimed to investigate whether an acute sleep restriction protocol (3 hours of sleep curtailment, either in the morning or in the evening) impaired EF by increasing impulsive behavior (in the attentional, motor and cognitive dimensions). We hypothesized that the morning restricted group would display the highest performance impairment, given that, unlike the evening restriction group, these individuals did not have the opportunity to compensate for the lack of sleep by engaging in regulatory mechanisms to enhance homeostatic sleep pressure dissipation.

## MATERIALS AND METHODS

### Participants

A total of 58 healthy young adults, aged 18-35 years old and non-smokers, took part in the present study. However, due to actigraph malfunction, data from only 48 subjects were suitable to be analyzed.

During the screening process, participants using psychotropic medication; with diagnosed sleep, psychiatric and/or neurological disorders, self-reported by the volunteers; chronotype defined as extreme morning or evening types and BMI classified as obese II/III^30^ were excluded. Additionally, volunteers that did not comply with the experimenter’s instructions (average weekly onset time later than 3 A.M; average weekly sleep duration minor than 3 hours; compensation of sleep restriction by sleeping 30 minutes or more; sleep restriction lesser than at least 90 minutes comparing to their weekly sleep duration average) or reported health problems on the day of the experiment were also excluded.

This study was approved by the local ethics committee and all participants signed written informed consent.

### Experimental design

This study aimed to mimic a real life scenario. Hence, a shorter and acute sleep restriction is more common daily experience than a TSD. Therefore, participants were randomly assigned to one of three groups: control, in which participants initiated sleep and woke up at their habitual time; morning restriction, in which volunteers woke up approximately three hours earlier than usual on the day of the experiment; evening restriction, in which volunteers initiated sleep approximately three hours later than usual in the night that preceded the experiment (experimental night). The volunteers had their sleep/wake cycle monitored by actigraphy (Basic Motionlogger-L Actigraph^®^, Ambulatory Monitoring, Inc., Ardsley, NY, United States) during the entire week preceding the experiment.

**Figure 1:**
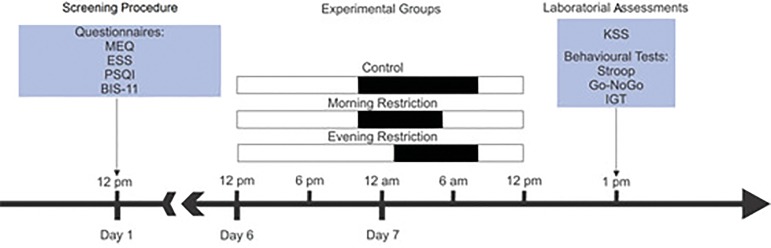
Study protocol. One week before the experimental day, the screening of volunteers through questionnaires (MEQ = Horne and Ostberg Morningness-Eveningness Questionnaire; ESS = Epworth Sleepiness Scale; PSQI = Pittsburgh Sleep Quality Index and BIS-11 = Barratt Impulsivity Scale) and the actigraphy recordings were initiated. On Day 6, volunteers were assigned to one of three groups: control, in which participants initiated sleep and woke up at their habitual time; morning restriction, in which volunteers woke up approximately three hours earlier than usual on the day of the experiment; evening restriction, in which volunteers initiated sleep approximately three hours later than usual in the experimental night (the white bars represent wakefulness and the black bars represent the sleep period). On the experimental day, they filled out the Karolinska Sleepiness Scale (KSS) and performed three behavioral tests: Stroop Test, Go-NoGo and Iowa Gambling Task (IGT).

In order to replicate a scenario as close to reality as possible, during the week prior to the experiment all participants continued following their usual routine, including sleeping at home. Only at midday of the experiment day the volunteers arrive at the laboratory, when they were debriefed about their health status by a questionnaire and performed three cognitive tests (described below) (Fig. 1). Besides that, all the volunteers were instructed to not consume caffeine, alcohol and other drugs in the day before the experimental night.

### Questionnaires and behavioral tests

The volunteers answered the Horne and Ostberg Morningness-Eveningness Questionnaire (MEQ)^31^ for assessing their chronotype; the Epworth Sleepiness Scale (ESS)^32^ to evaluate their trait daytime sleepiness; a health habits questionnaire, to evaluate health conditions including questions about previous diagnosed disorders; the Pittsburgh Sleep Quality Index^33^ and the Barratt Impulsivity Scale^34^, which was used to balance impulsivity traits among groups. In addition, subjects answered the Karolinska Sleepiness Scale (KSS)^35^ to assess their sleepiness state and then performed three behavioral tests: the IGT, the Stroop Test, and the Go-NoGo test, in this order.

The Victoria version of Stroop Test^36^, performed manually, was used to assess selective attention. The goal was to name the printed colors as quickly as possible. The test consists of three cards measuring 20 cm X 30 cm, with 24 stimuli arranged in four column: the first card had painted rectangles; the second card had neutral words (unrelated to concepts of color: ‘each’, ‘never’, ‘today’, ‘everything’) printed in the same colors of the first card; the last one, called interference card, had the names of colors as stimuli (brown, blue, pink and green), but the printing ink color never matched the color name. The time to complete each card was clocked and could not exceed 120 s. Regarding the interference card, the variables analyzed were: time to complete the interference card and interference index, calculate through the following formula:

$$\mathrm{Interference}\;\mathrm{Index}=\frac{\mathrm{TR}1+\mathrm{TR}2}{2}=\mathrm{TR}3$$

Where TR1 means the time to respond the card 1, TR2, time to respond the card 2, and TR3, time to respond the card 3. This measure is described as the increase in time spent to accomplish the task in the presence of distractor stimuli.

To assess inhibition of motor response, a computerized version of the Go-NoGo test was applied. A total of 180 stimuli were presented and different stimuli demanded different responses: GO stimuli (green rectangles), required that the volunteers to press a button, and NOGO stimuli (orange rectangles), in which the volunteers should not press the button. The stimuli were displayed with a frequency of 75% and 25%, respectively, and remained on the screen for 200ms. The variables analyzed were the number of false alarms (failure to inhibit the response against the NOGO stimulus), number of correct responses, number of errors (not pressing the button in the GO stimulus) and reaction time when answered correctly.

The IGT assesses the ability to modify decision making strategies based on implicit learning of punishment and reward contingencies^37^. The task was presented in a computerized version and was performed in 100 trials. In each one, participants had the possibility of choosing between four decks of cards (A, B, C, D) with a pattern to monetary losses and gains (fictitious): two decks containing cards of greater rewards and a high probability of large losses (called disadvantageous decks); and two decks containing cards of smaller rewards and low probability of large losses (advantageous decks). The amount of fictitious money won/lost for each trial was visible on the screen, as well as the accumulated balance. It was informed to the participants that the goal of the task was to win as much money as possible, that they could switch the deck in every trial and that there was no pre-established limit of time to complete the task. Performance was measured by the total won and the number of selected advantageous cards.

### Statistical analysis

Gaussian distribution was verified with a Shapiro-Wilk’s test. Variables with normal distribution were expressed as mean ± standard deviation (SD) while non-parametric variables were expressed as median (minimum-maximum). Comparisons between groups regarding sample characterization and actigraphy were made using one-way ANOVA and Tukey’s *post hoc* test. The EF tests’ variables and KSS were compared using the Kruskal-Wallis test, outliers were identified and excluded by means of Rout’s test performed and Dunn method was performed when necessary in Prisma version 6 GraphPad Software. *p* values < 0.05 were considered statistically significant.

## RESULTS

### Sample characteristics

The experimental groups did not differ in age (F=1.96; *p*=0.15), BMI (F=0.48; *p*=0.95), scores on the Barratt Impulsiveness Scale (F=0.67; *p*=0.51), Horne and Ostberg questionnaire (F=0.55; *p*=0.57) and Epworth Sleepiness Scale (F=1.82; *p*=0.17). A chi-square analysis did not identify any significant difference in sex distribution among groups (*p*=0.18). Data are presented in Table 1.

**Table 1:** Sample characterization.

	Control	Morning restriction	Evening restriction	F; *p*
n (M/F)	18 (5/13)	17 (10/7)	13 (5/7)	-
Age	23.2 (4.8)	21.3 (4.9)	24.5 (3.4)	1.96; 0.15
BMI	23.2 (3.7)	23.2 (2.7)	22.9 (2.8)	0.48; 0.95
BIS-11	65 (10.5)	63 (8.9)	61 (7.2)	0.67; 0.51
MEQ	49.7 (8.5)	52.8 (7)	51.1 (10.7)	0.55; 0.57
ESS	9.2 (3.02)	7.5 (3.4)	6.9 (4.5)	1.82; 0.17

Means (SD); N=sample size (males\females); BMI= body mass index; BIS-11=Barratt Impulsiveness Scale; MEQ=Horne & Ostberg questionnaire; ESS=Epworth Sleepiness Scale. *p* values depict the result of a one-way ANOVA.

### Sleep patterns

To confirm the participants’ compliance with the specific instructions given to each group, we compared actigraphy variables from both the entire week and the experimental night by a one-way ANOVA.

Regarding the comparison during the week prior to the experimental day, no significant differences across groups were found in sleep onset time (F=0.99; *p*=0.37), sleep offset time (F=0.7; p=0.5) and sleep duration (F=1.68; *p*=0.19). Consistent with the instructions for sleep restriction condition, when evaluating the experimental night, we found significant differences across groups in sleep onset time (F=18.14; *p*<0.001), sleep offset time (F=29.51; <0.001) and sleep duration (F=25.54; <0.001).

Tukey’s *post hoc test* showed that on the experimental night the evening group initiated sleep later than the control (*p*<0.001) and morning (*p*<0,001) groups, while the morning group woke up earlier than the control (*p*<0.001) and evening (*p*<0.001) groups. As expected, both morning and evening restricted groups displayed shorter sleep duration than the control group (*p*<0.001 and *p*<0.001, respectively). Descriptive statistics of actigraphy variables is summarized in Table 2.

**Table 2:** Sleep patterns.

	Control	Morning restriction	Evening restriction	F; *p*
Weekly onset time	12:39 A.M (60)	12:22 A.M (59)	12:08 A.M (59)	0.99; 0.37
Weekly offset time	8:10 A.M (48)	8:19 A.M (60)	7:54 A.M (66)	0.70; 0.50
Weekly sleep duration	6h24 (53)	6h58 (54)	6h38 (61)	1.68; 0.19
Sleep onset time prior to experiment day	12:32 A.M (87)	12:28 A.M (71)	02:53 A.M (47)	18.14; <0.001
Sleep offset time prior to experiment day	8:09 A.M (80)	4:44 A.M (92)	7:25 A.M (66)	29.51; <0.001
Sleep duration prior to experiment day	6h32 (87)	3h49 (68)	4h05 (57)	25.54; <0.001

Values represented as mean (SD, in minutes). *p* values depict the result of a one-way ANOVA.

### Subject sleepiness

On the experimental day, the participants’ subjective sleepiness was assessed through the KSS and no significant differences across groups were found (H=2.22; *p*=0.32).

### Executive functioning

**Figure 2:**
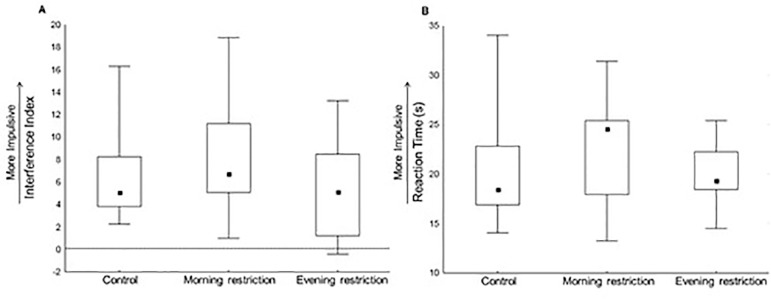
Selective attention as assessed by the Stroop Test. A: interference index; B: reaction time; Control: n=17; Morning-restriction: n=17; Evening-restriction: n=13. Performance was compared across groups by a Kruskal-Wallis test. No statistical difference was found. Values are represented as median (minimum/maximum).

The interference index (H=3.38; *p*=0.18) and the time to perform the interference card (Reaction Time) (H=2.61; *p*=0.27) on the Stroop Test, used to assess selective attention, did not differ across groups (Fig. 2).

**Figure 3:**
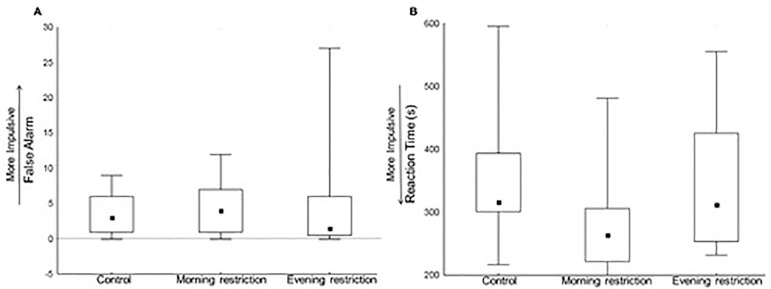
Motor inhibition as assessed by the Go-NoGo Test. A: number of false alarm (Control: n=18; Morning-restriction: n=17; Evening-restriction: n=13); B: reaction time (Control: n=18; Morning-restriction: n=17; Evening-restriction: n=13). Performance was compared across the three sleep conditions by a Kruskal-Wallis test with Dunn method. No statistical difference was found between groups in the post hoc test. Values are represented as median (minimum/maximum).

Regarding the Go-NoGo Test, the number of false alarms (H=0.8; *p*=0.67) did not differ across groups, but there was a significant effect in reaction time (H=6.30; *p*=0.04) (Fig. 3). However, this effect did not survive *post hoc* comparisons (Control *vs.* Morning *p*=0.57; Control *vs.* Evening *p*=1.0; Morning *vs.* Evening *p*=0.17).

**Figure 4:**
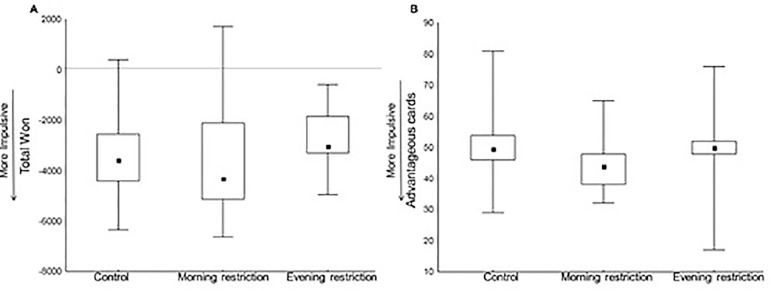
Decision-making ability as assessed by the Iowa Gambling Task. A: total won; B: number of advantageous selected cars. Control: n=18; Morning-restriction: n=17; Evening-restriction: n=13. Performance was compared across the three sleep conditions by a Kruskal-Wallis test. No statistical difference was found. Values are represented as median (minimum/maximum).

The ability to make decisions, assessed by the IGT, was compared using the total won (H=2.64; *p*=0.26) and the number of selected advantageous cards (H=4.43; *p*=0.11), and no significant differences were found across the three sleep conditions (Fig. 4).

## DISCUSSION

The aim of this study was to evaluate if an acute sleep restriction protocol could affect EF by impairing the three dimensions of impulsive behavior (attentional, motor and cognitive) in healthy young adults. We have shown that following one night of 3 hours of sleep restriction, selective attention, response inhibition and decision making abilities persisted unaffected, regardless of when the restriction occurred: in the SWS-rich first night half or in the REM-rich late night half. In other words, our results suggest that the mechanisms involved in homeostatic regulation of sleep pressure in the nervous system might be able to sustain executive functioning within its normal range even after 3 hours of sleep restriction.

The actigraphy data showed that the sleep-restricted groups had, in fact, a shorter sleep duration if compared to the control group. However, our results indicate that this reduction in sleep duration was not reflected by increased daytime sleepiness, as assessed by the KSS. Since the KSS was originally intended to be employed following long periods of sleep deprivation, it might not be a very accurate tool to detect more subtle changes in subjective sleepiness as in the present study.

It is well established that decreasing sleep duration negatively impacts cognitive functions such as attention and motor performance, besides increasing feelings of fatigue and drowsiness^38^. However, those effects were observed following sleep deprivation periods larger than 24 hours. Healthy young adults exposed to more than 40 hours of wakefulness had an impairment in selective attention assessed by the Stroop Test, showing increased time to respond to the interference card but an unaffected interference index^20^. However, there is still no consensus concerning the effects of sleep loss when evaluating different versions of the Stroop Test, since after 36 hours of waking, Sagaspe et al.^19^ did not find any impairments on participants’ performance.

Impairments in the ability to inhibit a motor response as assessed by the Go-NoGo Test were found after 24^21^ and 55 hours^22^ of wakefulness denoted by an increased number of false alarms and a decreased number of correct responses. Similarly, decision making abilities as assessed by the IGT were significantly impaired after 40 hours of wakefulness in healthy young adults^24^. Furthermore, there are indications in the literature of a relationship between increased sleepiness, sleep deprivation and impaired decision-making^25^.

Simulations of chronic sleep restriction were shown to also affect cognitive faculties. Women exposed to 4 hours of sleep restriction for three consecutive nights showed increased response time and number of errors when performing the interference card on the Stroop Test, independent of their age group (young: 20 - 30 years old; aged: 55 - 65 years old)^39^. In addition, exposure to 3 hours of sleep restriction for 4 nights resulted in higher numbers of false alarms on the Go-NoGo Test in healthy middle age adults (mean age=37 years old)^40^.

Since the protocol used in our study exposed participants to 3 hours of acute sleep restriction, a comparison with studies of chronic sleep restriction or total sleep deprivation is not straightforward. However, our results are in line with evidence from a study with a similar acute sleep restriction protocol (4 hours, early in the morning), which also did not find impaired performance on the GoNo test in young adults^41^.

Sleep duration in the sleep-restricted groups was 4h 30min on average, compared to 6h 30min on average in the control group. According to the core sleep hypothesis, a normal nocturnal sleep period is comprised of two types of sleep: core sleep, the initial sleep period capable of repairing the effects of the wake period, and optional sleep, all sleep obtained beyond the core sleep^42^. It has been postulated that only the core sleep, especially the portion dominated by slow wave activity, is necessary for adequate daytime functioning, whereas optional sleep does not contribute to it, being more liable to variate in duration than the first portion with a few or even no consequences in cognition^42^.

The core sleep duration was placed at 4 to 5 hours of sleep per night^42^, which corresponds approximately to the sleep duration available to our sleep deprived sample, thereby explaining the absence of cognitive impairments. However, the circadian variation in REM sleep also needs to be taken into account^43^. The evening group probably had greater REM sleep duration in detriment of NREM sleep duration, when compared to the two other groups. If so, the evening group should have been more affected by the sleep restriction protocol. Nonetheless, these subjects spent more time awake before sleep onset, that is, they have accumulated more sleep pressure. EEG studies show that the more the time spent awake before sleep onset, the highest the delta and slow wave activity (SWA) power during SWS^44^.

Therefore, unlike the morning group, the evening group could have compensate for a shorter overall sleep duration by potentiating sleep pressure dissipation with increased delta/SWA power and higher slow oscillation (SO) amplitude and/or slope, the typical features of rebound sleep. On the other hand, even though the Morning Restriction group did not have the opportunity to engage in rebound sleep, core sleep might have been preserved. However, those speculations could not be verified since the experimental night was not under a controlled environment (e.g. in the lab) along with a polysomnography exam to monitor sleep architecture.

It is known that TSD has an adverse effect on processes that involve activity in the prefrontal cortex, such as planning, judging and deciding^45^. The decreased metabolic rate after TSD throughout the prefrontal cortex observed in adults^26^ could explain the impairments in attention, motor inhibitory response and decision making^27^^-^^29^. When measuring the effects of 35 hours of sleep deprivation on cerebral activation during verbal learning, the prefrontal cortex was more responsive after TSD than after normal sleep^46^, a finding also observed when performing a divided attention task, where the parietal lobes and cingulate gyrus were more responsive after TSD^47^. These data suggest a possible compensatory mechanism occurring after sleep deprivation to ensure executive functioning. Although it is not capable of compensating impairments in executive functions after long periods of sleep deprivation, it seems effective in overcoming the homeostatic sleep pressure accumulated following a sleep restriction period.

Recently, it has been demonstrated that cortical timescales at the individual neuron-level in rats change as a function of vigilance state and time awake. In situations in which timescales and information integration are not affected by intermittent high amplitude events, normal performance would still be feasible in a situation of sleep restriction^48^.

The sensitivity of executive functions probing tests should also be considered when interpreting our results. Tucker et al. suggest that the lack of an effect in a sleep deprivation protocol upon executive functions could be due to the non-executive components contained in each test. Sustained-attention, one of several cognitive elements used to execute an action, is more required in some executive functions tests than others and this cognitive domain is hardly deteriorated after sleep deprivation^49^^,^^50^. Most of the neuropsychological instruments available were developed for lesioned patients, e.g. the IGT, which was tested firstly in patients with damage to the ventromedial prefrontal cortex^37^. Therefore, since this kind of test has been employed mostly in conditions with highly impaired cognitive functions, it is possible that it is not sensitive enough to assess lighter impairments such as those due to acute sleep restriction.

The effect of sleep deprivation on performance in neurocognitive tasks can be vulnerable to individual differences^51^. Thus, a limitation of the present study is that our experimental design did not allow for within subject comparisons. Besides in the present study, sleep was not monitored via polysomnography during the sleep restriction night, which could have provided essential information concerning individual differences in homeostatic sleep pressure compensation mechanisms during sleep restriction.

In conclusion, after exposure to a protocol of 3 hours of acute sleep restriction, in which subjects were free to engage in work or leisure activities as they pleased, the ability to change strategies and make decisions, focus on a given task and respond to unforeseen challenges is preserved in healthy young adults. We believe that our experimental design contributes to more closely resembling the ordinary scenario that most people are commonly faced with: a curtailment in sleep duration due to work, social or personal obligations followed by a regular day of work where they are expected to keep performance at the highest level. Thus, to preserve executive functioning, it should be better to shorten only a few hours instead of a whole night.
